# Reversal of Bortezomib Resistance in Myelodysplastic Syndrome Cells by MAPK Inhibitors

**DOI:** 10.1371/journal.pone.0090992

**Published:** 2014-03-07

**Authors:** Yingxing Yue, Ying Wang, Yang He, Shuting Yang, Zixing Chen, Yuanyuan Wang, Shanshan Xing, Congcong Shen, Hesham M. Amin, Depei Wu, Yao-Hua Song

**Affiliations:** 1 Cyrus Tang Hematology Center, Jiangsu Institute of Hematology, First Affiliated Hospital, Soochow University, Suzhou, China; 2 Jiangsu Institute of Hematology, The First Affiliated Hospital of Soochow University, Key Laboratory of Thrombosis and Hemostasis of Ministry of Health, Suzhou, China; 3 Department of Hematopathology, The University of Texas MD Anderson Cancer Center, Houston, Texas, United States of America; Northwestern University, United States of America

## Abstract

The myelodysplastic syndromes (MDS) comprise a heterogeneous group of malignant neoplasms with distinctive clinicopathological features. Currently, there is no specific approach for the treatment of MDS. Here, we report that bortezomib (BTZ), a proteasome inhibitor that has been used to treat plasma cell myeloma, induced G2/M phase cycle arrest in the MDS cell line SKM-1 through upregulation of Wee1, a negative regulator of G2/M phase transition. Treatment by BTZ led to reduced SKM-1 cell viability as well as increased apoptosis and autophagy. The BTZ-induced cell death was associated with reduced expression of p-ERK. To elucidate the implications of downregulation of p-ERK, we established the BTZ resistant cell line SKM-1R. Our data show that resistance to BTZ-induced apoptosis could be reversed by the MEK inhibitors U0126 or PD98059. Our results suggest that MAPK pathway may play an important role in mediating BTZ resistance.

## Introduction

The myelodysplastic syndromes (MDS) are a group of clonal disorders characterized by ineffective hematopoietic cell production and variable risk of transformation to acute myeloid leukemia (AML). Treatment options are limited and targeted therapies are not available for MDS. Hematopoietic stem cell transplantation (HSCT) strategies may improve long-term survival in some young patients. However, MDS is primarily a disease of elderly people who are often intolerant to aggressive therapies such as HSCT and chemotherpeutics.

It has been shown that the proteasome inhibitor bortezomib (BTZ) is effective in the treatment of plasma cell myeloma [1] [2] [3]. More recently, BTZ demonstrated some promise in the treatment of MDS and AML [4]–[7]. In a phase I clinical trial, BTZ combined with weekly idarubicin successfully induced hematologic response in AML patients who have prior history of MDS [5]. Similarly, in a phase I/II trial, BTZ and low dose cytarabine arabinoside showed clinical response in 36% of high-risk MDS patients [7]. These studies also demonstrated that BTZ is more effective when combined with other chemotherapeutic agents for treating high-risk MDS patients [5] [7]. Nonetheless, chemotherapy is usually associated with severe side effects that might lead to patient’s death. Most likely, targeted therapies that selectively exploit specific survival molecules are more effective and notably associated with fewer side effects. The development of targeted therapies for MDS has been particularly challenging due to the complexity of the oncogenic systems contributing to the survival of MDS cells.

The MEK/ERK pathway plays key roles in controlling cell survival and cell cycle progression, and its deregulation is often implicated in developing drug resistance and cancer progression. Upregulation of p-ERK has been observed in the majority of AML cases [8], [9], and elevated expression of ERK in AMLs is associated with a poor prognosis [10]. Furthermore, introduction of a constitutively activated form of MEK into hematopoietic stem cells causes myeloid malignancies such as MDS and myeloproliferative neoplasms [11]. Persistant activation of MEK/ERK pathway mediates drug resistance in leukemia cells [12]–[15]. These studies suggest that MEK/ERK pathway may play a role in the development of MDS and in mediating drug resistance.

In this study, we investigated the effects of BTZ in a human MDS cell line SKM-1. Our results demonstrated that p-ERK1/2 is highly expressed in SKM-1 cells. The expression of p-ERK1/2 was markedly decreased after treatment with BTZ. In contrast, treatment with BTZ resulted in upregulation of ERK in the BTZ-resistant cell line SKM-1R. However, the resistance to BTZ in SKM-1R cells was reversed by the MEK inhibitors U0126 and PD98059. This study provides the first evidence that MEK/ERK pathway mediates BTZ resistance and suggests that MEK/ERK inhibitors could be successfully used in conjunction with BTZ to overcome drug resistance in MDS.

## Materials and Methods

### Cell Culture and Reagents

The human MDS cell line SKM-1 has been described previously [16]. SKM-1 cells were maintained in RPMI −1640 with 20% fetal calf serum (HyClone), 100 U/ml penicillin and 100 µg/ml streptomycin in 5% CO_2_ at 37°C. The BTZ-resistant SKM-1 cell line was established by repeated exposure of the cells to 5 nM of BTZ for 24 hours followed by 2 weeks recovery over a period of 3 months. MEK inhibitors PD98059 and U0126 were purchased from Cell Signaling Technology.

### MTT Assay

Cell viability was assessed by the MTT assay. MTT reagent was purchased from Sigma. Human SKM-1 cells were treated with BTZ in 96 well plates at the density of 2×10^4^/well in each experiment. After 24 h, MTT assay was performed. The absorbance was measured at 490 nm by a micro-plate reader (Spectra Max M5).

### Measurement of Apoptosis and Cell Cycle

Apoptosis was assessed by flow cytometry (FACS Calibur Flow Cytometer, BD Biosciences) for Annexin V and propidium iodide (PI) staining (kit from Roche). Cells that are positive for Annexin V but negative for PI are considered undergoing apoptosis. Cell cycle analysis was performed by flow cytometry for PI staining (Sigma).

### Immunoblotting

SKM-1 cells were washed with PBS and then lysed in a buffer containing 20 mM HEPES (pH 7.2), 10% glycerol, 150 mM NaCl, 1%TritonX-100, 50 mM NaF, 1 mM Na3VO4, 10 ug/ml leupeptin, 10 ug/ml aprotinin, and 1 mM PMSF. Proteins were separated by SDS–polyacrylamide gel electrophoresis and blotted onto a PVDF membrane (MILLIPORE). Primary antibodies (Cell Signaling Technology) were directed against Wee1 (1∶1000), cdc25C (1∶1000), p-ERK (1∶1000), ERK (1∶2000), LC3 (1∶2000), cleaved caspase-3 (1∶1000) and GAPDH (1∶5000). Secondary antibodies were anti-rabbit IgG (1∶5000, Cell Signaling) and anti-mouse IgG (1∶5000, Sigma). Antigen–antibody complexes were visualized by enhanced chemi- luminescence (PerkinElmer).

### Immunofluorescence

Cells were fixed with 4% paraformaldehyde and incubated with blocking buffer containing 1% BSA and 0.1% Triton X-100. After blocking, cells were incubated overnight in the primary antibody (cleaved caspase-3, 1∶400). After washing, cells were incubated in goat anti-rabbit IgG-Alexa Fluor R 568 (Molecular Probes, diluted 1∶500). Cells were then rinsed and counterstained with DAPI. Images were taken by fluorescence microscope (Leica DM2000).

### Statistical Analysis

Data are presented as means ± SD. Student t test was used to determine the significance of the differences between variables. P<0.05 was considered statistically significant.

## Result

### BTZ Causes Cell Cycle Arrest in G2/M Phase via Increasing Wee1 Expression

**Figure 1:**
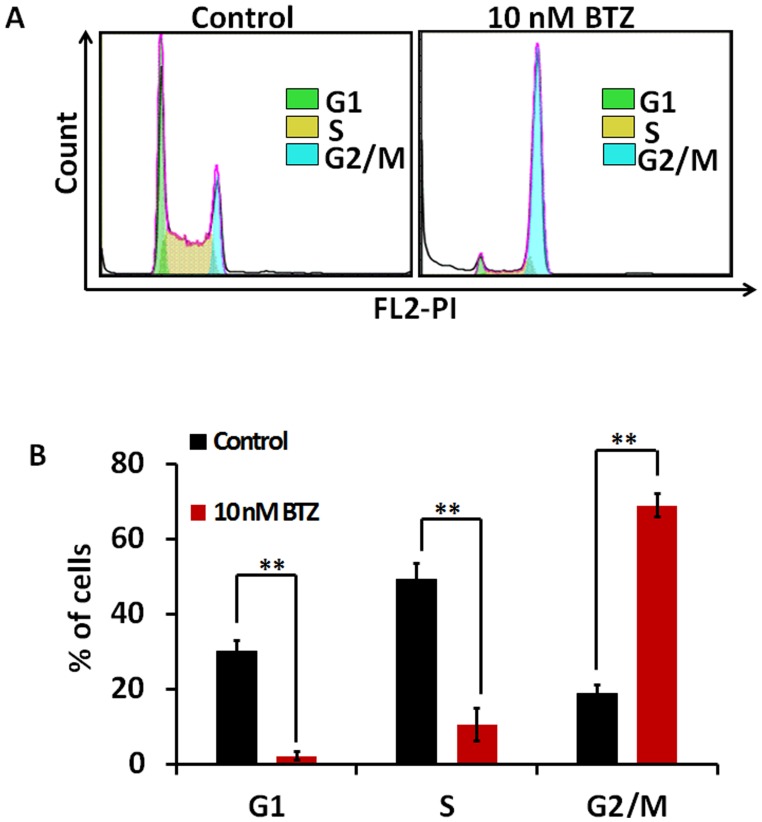
BTZ arrests SKM-1 cells at G2/M phase. A. SKM-1 cells were treated with vehicle (control) or 10 nM BTZ for 24 h and then stained with PI for FACS analysis. B. The distribution of cells in each cell cycle phase is shown. **P<0.01.

The effect of BTZ on cell cycle progression in SKM-1 cells was evaluated using flow cytometric analysis. After treatment with 10 nM BTZ for 24 h, the percentage of cells in G1 and S phases decreased 28% and 29% respectively, whereas the percentage of cells in G2/M phase increased 51% (Fig. 1). Our results indicate that BTZ is able to arrest SKM-1 cells at G2/M.

**Figure 2:**
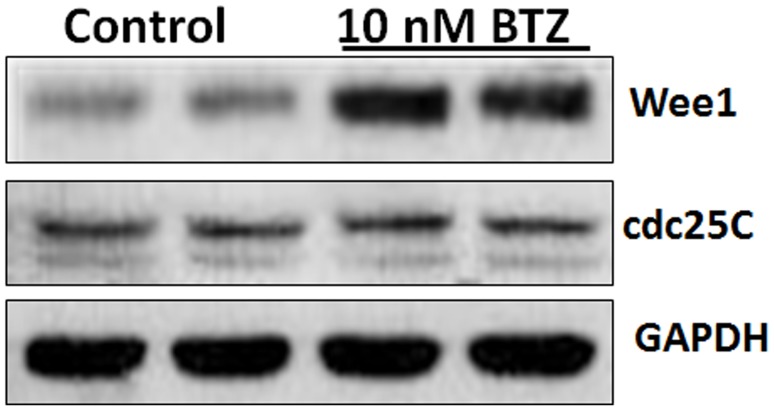
Effect of BTZ on the expression of cell cycle regulatory proteins Wee1 and cdc25C. SKM-1 cells were treated with 10 nM BTZ and the expression of Wee1 and cdc25C was analyzed by immunoblotting. GAPDH was used as loading control.

Entry of eukaryotic cells into M-phase of cell cycle is controlled by cdc2 kinase which is activated by cdc25C and inactivated by Wee1. At the G2/M transition, Wee1 is downregulated while cdc25C is upregulated [17]. The finding of a G2/M arrest by BTZ led us to examine the expression levels of Wee1 and cdc25C. Fig. 2 shows that treatment with 10 nM BTZ for 24 h resulted in increased Wee1 protein expression, while the protein level of cdc25C was not altered. These data suggest that increased Wee1 protein expression contributes to cdc2 inhibition and subsequent G2/M cell cycle arrest following BTZ treatment. Previous studies have shown that cell cycle arrest at G2/M phase often leads to induction of apoptosis and cell death [18].

### BTZ Induced Cell Death in SKM-1 Cells is Mediated by Down-regulation of p-ERK

**Figure 3:**
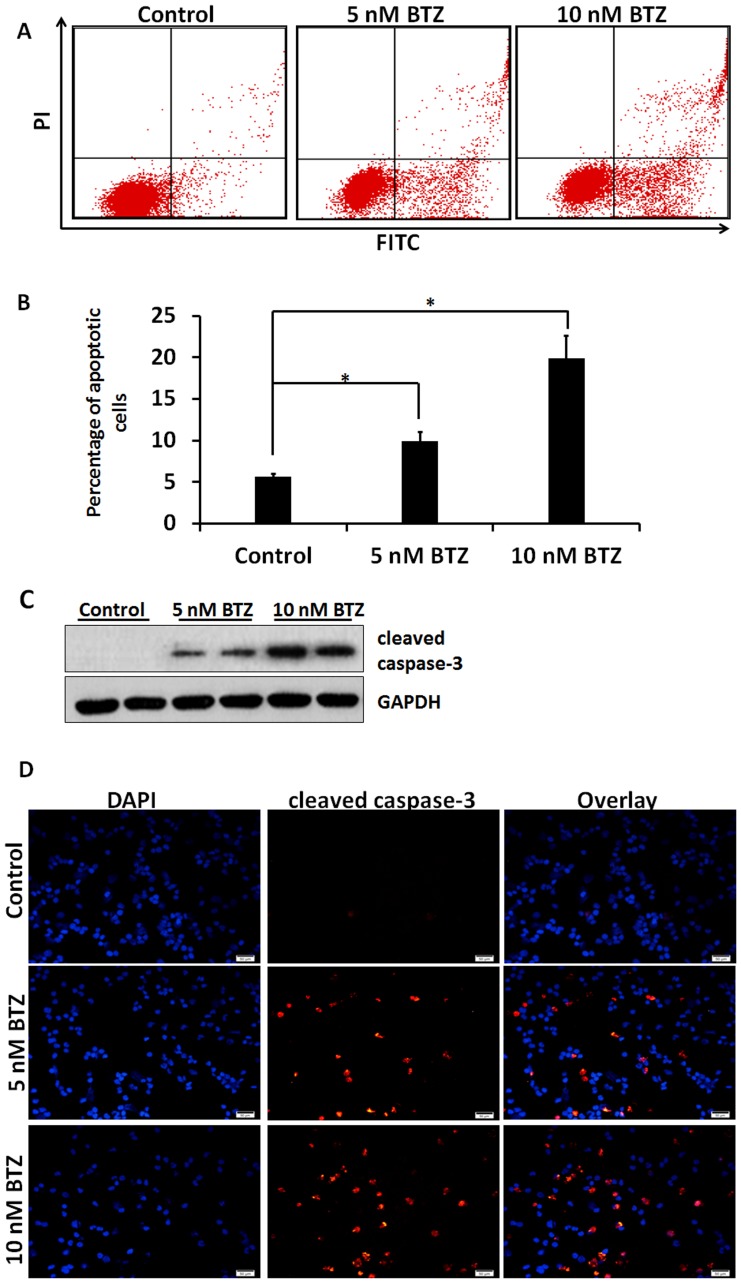
BTZ induces apoptosis. A. Representative flow cytometry scatter plots showing cells undergoing apoptosis in response to BTZ. B. Bar graph shows percentage of SKM-1 cells undergoing apoptosis in response to 5 and 10 nM BTZ for 24 h. C. Immunoblot analysis of cleaved caspase-3. D. Immumofluorescence staining of cleaved caspase-3. Bar 50 µm. *P<0.05.

Recent studies showed that BTZ induced apoptosis in plasma cell myeloma cells [19], [20]. The ability of BTZ to induce apoptosis in SKM-1 cells was evaluated by flow cytometry. As shown in Fig. 3A and 3B, at 24 h, BTZ caused an increase of the percentage of apoptotic cells in a concentration-dependent manner. Immunoblot analysis and immunofluorescence staining of cleaved caspase-3 further confirmed apoptosis (Fig. 3C,D).

**Figure 4:**
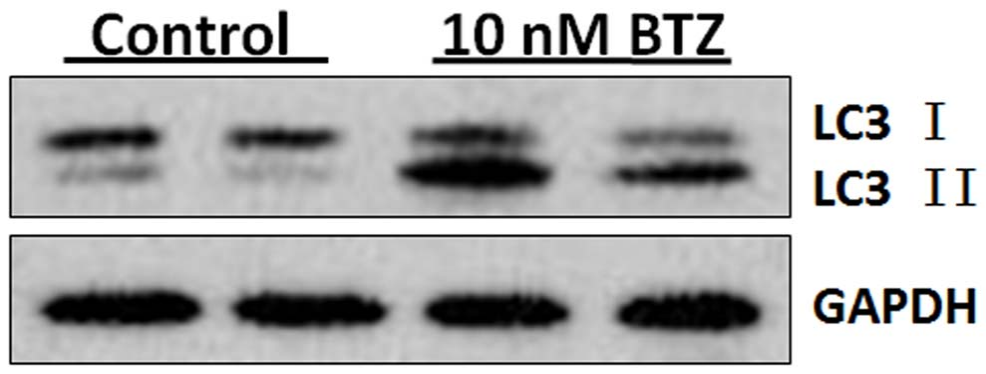
BTZ induces autophagy in SKM-1 cells. SKM-1 cells were treated with 10 nM BTZ for 24 h and immunoblotted for microtubule-associated light chain (LC3-I and LC3-II). GAPDH was used as loading control.

It has been shown that BTZ has the capacity to induce autophagic cell death in MDS cells [21]. The induction of autophagy was evaluated in the SKM-1 cells. LC3-II has been used as a marker for autophagy. During induction of autophagy, LC3-I present in the cytoplasm is converted to LC3-II which is membrane bound and correlated with the extent of autophagosome formation [22]. As shown in Fig. 4, an increase of LC3-II levels is evident in BTZ treated cells.

### MAPK Inhibitors Overcome BTZ Induced Drug Resistance

**Figure 5:**
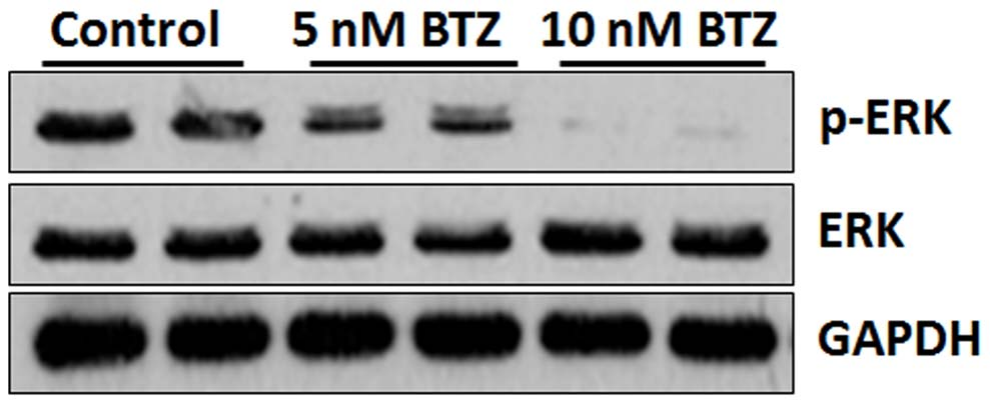
ERK1/2 expression in SKM-1 cells. Immunoblotting of SKM-1 cells shows that total and p-ERK1/2 are both expressed in untreated SKM-1 cells (control). However, BTZ inhibited the expression of p-ERK1/2 but not total ERK1/2. GAPDH was used as loading control.

Given the established role of MAPKs in controlling cell survival, we evaluated if MAPK pathway could be involved in BTZ induced apoptosis. The expression of p-ERK1/2 and total ERK1/2 was analyzed by immunoblotting. SKM-1 cells show constitutively high expression of p-ERK/ERK (Fig. 5). Treatment with BTZ dramatically suppressed the expression of p-ERK1/2 but not total ERK1/2 at 24 h (Fig. 5).

**Figure 6:**
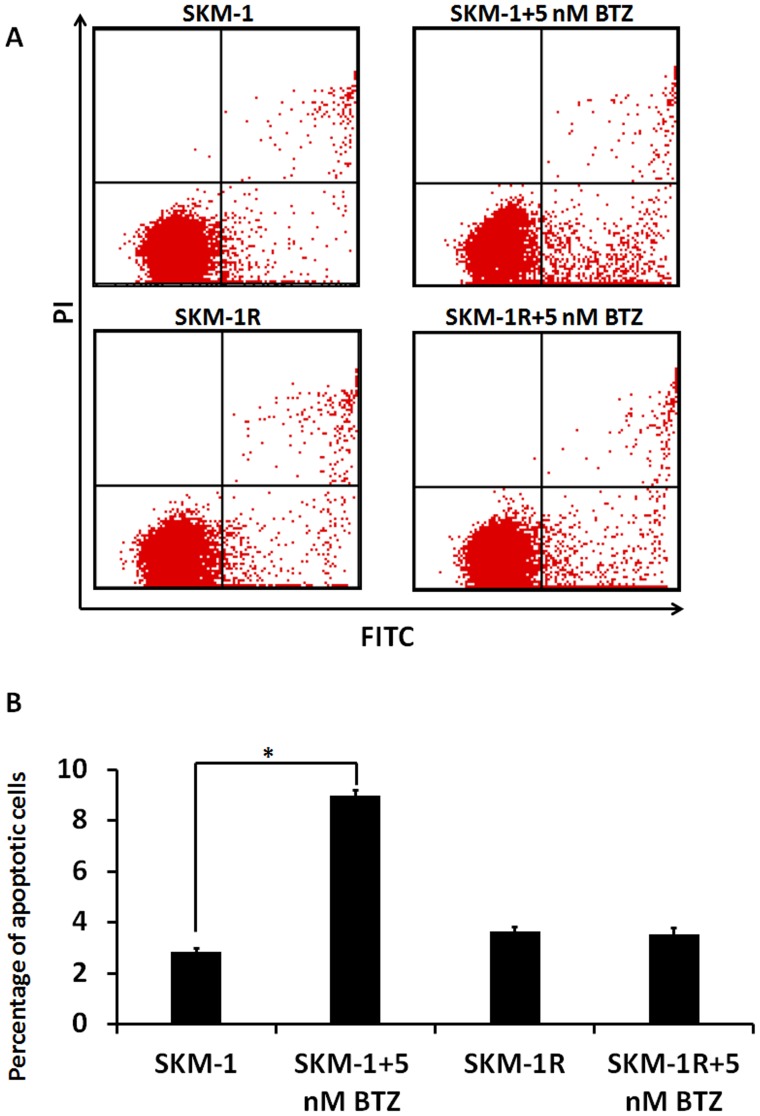
SKM-1R cells are resistant to BTZ induced apoptosis. A. Flow cytometry scatter plots of the apoptosis assay. B. Bar graph shows percentage of SKM-1 and SKM-1R cells undergoing apoptosis in response to a 24 h treatment with 5 nM BTZ. *P<0.05.

**Figure 7:**
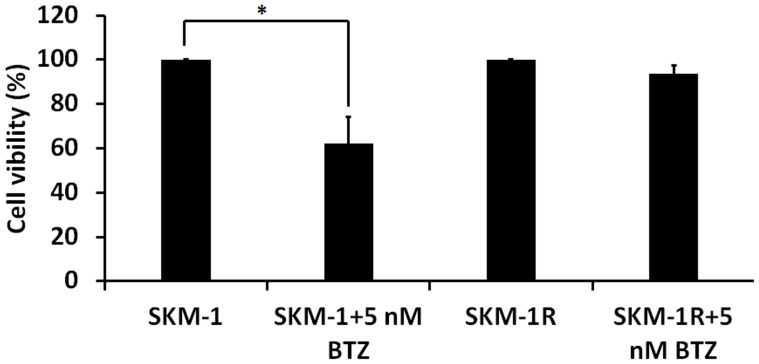
Cell viability in response to BTZ treatment. SKM-1 and SKM-1R cells were exposed to 5 nM BTZ for 24 h and the viable cell number was evaluated by MTT assay. The result showed a reduction in cell viability by treating wild type SKM-1 cells with 5 nM BTZ with no notable effect on SKM-1R cells. *P<0.05.

**Figure 8:**
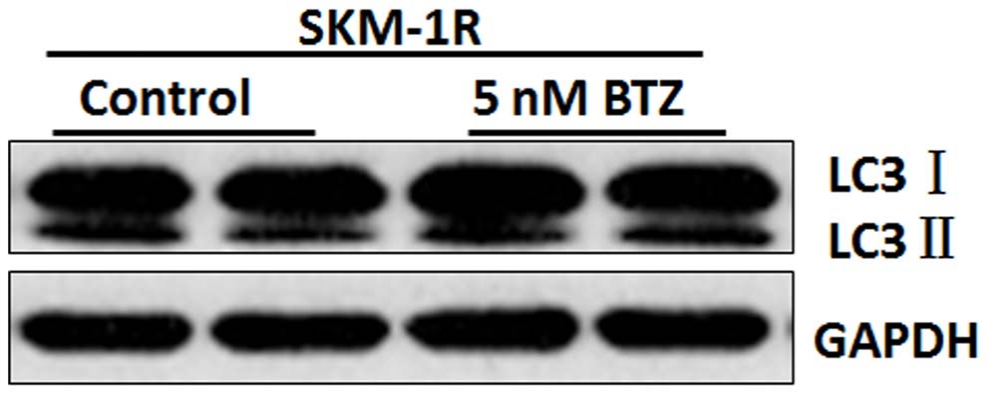
Expression of LC3 in BTZ resistant SKM-1R cells. Cell lysates were subjected to immunoblot analysis using specific antibody against LC3. Conversion of LC3-I to LC3-II was not observed in SKM-1R cells treated with BTZ.

**Figure 9:**
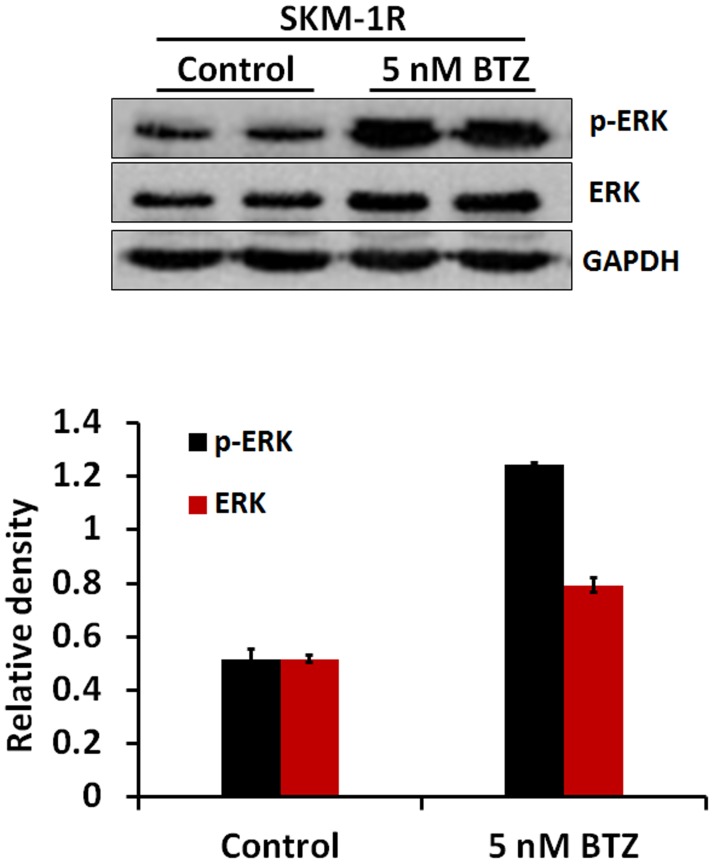
ERK1/2 expression in BTZ resistant SKM-1R cells. Immunoblotting of SKM-1R cells shows that both total and phospho-ERK1/2 are upregulated in response to BTZ treatment. Protein expression levels were quantified by ImageJ. Total and phospho-ERK1/2 levels were normalized to GAPDH.

Most patients who receive the first treatment with BTZ develop resistance to this drug [1]. In order to elucidate the mechanisms of drug resistance, we established a BTZ resistant cell line SKM-1R. Figure 6 demonstrated the induction of apoptotic cell death in SKM-1 cells treated with 5 nM BTZ by Annexin/PI staining. However, the percentage of cells undergoing apoptosis in BTZ treated SKM-1R cells was not different from the untreated SKM-1R cells. The observation that SKM-1R cells are resistant to BTZ induced apoptosis is consistent with our findings that cell viability did not decrease in BTZ treated SKM-1R cells (Fig. 7). Furthermore, activation of autophagy was not observed in SKM-1R cells treated with 5 nM BTZ (Fig. 8). We next evaluated the expression of ERK1/2 in SKM-1R cells. In contrast to wild type SKM-1 cells, the expression of both total and phospho-ERK1/2 was upregulated by BTZ in SKM-1R cells (Fig. 9).

**Figure 10:**
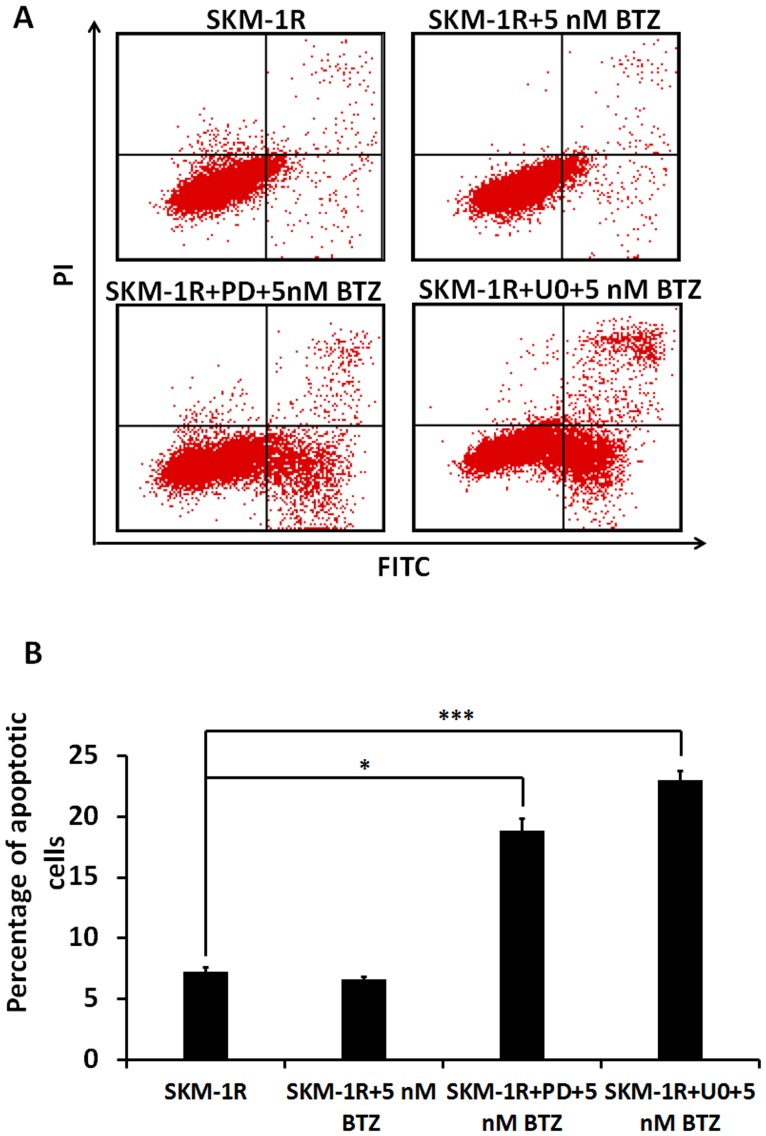
Reversal of BTZ resistance by MEK inhibitors in SKM-1R cells. A. Flow cytometry scatter plots showing Annexin V staining of SKM-1R cells. B. Bar graph shows percentage of SKM-1R cells undergoing apoptosis in response to a 24 h exposure to 5 nM BTZ. Both PD98059 (PD) and U0126 (U0) significantly increased the percentage of cells undergoing apoptosis. *P<0.05, ***P<0.001.

To further explore the role of MAPK pathway in the development of BTZ resistance in SKM-1R cells, the effects of MAPK inhibitors U0126 and PD98059 on BTZ-induced apoptosis were examined. Flow cytometry analysis showed that resistance to BTZ induced apoptosis was reversed by PD98059 and U0126 (Fig. 10A,B).

## Discussion

Despite the clinical success of BTZ in treating plasma cell myeloma, resistance to this drug remains a significant problem [1]. A phase 2 study showed that the rate of response to BTZ was only 35% and patients developed resistance to BTZ 12 months after the initial treatment [2]. A recent study showed that only 40% patients with relapsed plasma cell myeloma responded to the retreatment [3].

Significant progress has been made in elucidating molecular mechanisms of BTZ resistance. Oerlemans et al [23] found an Ala49Thr mutation residing in a highly conserved BTZ-binding pocket in the proteasome β5-subunit (PSMB5) protein in BTZ resistant human myelomonocytic THP1 cells. The mutation resulted in overexpression of the PSMB5 protein [23]. Upregulated expression of the PSMB5 gene was confirmed in bone marrow cells of multiple myeloma patients who developed BTZ resistance [24]. Ria et al [25] suggested that HIF-1α may also play a role in developing BTZ resistance by inducing angiogenesis in tumors. Relative few studies can be found in the literature regarding BTZ and AML. Most of these articles documented the effectiveness of BTZ for a subset of AML patients. BTZ resistant AML cell lines have not been established so far. However, it has been shown that persisting NF-κB activity may be responsible for the survival of CD34(+) AML cells after BTZ treatment [26]. When the IKK inhibitor BMS-345541 was used in combination with BTZ, the survival of CD34(+) AML cells was reduced [26], which suggest that NF-kB may be involved in BTZ resistance in AML. Bcl-2 overexpression has been suggested as a potential mechanism of BTZ resistance in human lymphoid cells [27]. Overexpression of heat shock proteins and T-cell factor 4 has been reported in BTZ resistant B-lymphoma cells [28]. Nonetheless, the molecular mechanisms/signaling pathways that mediate BTZ resistance in cancer cells including MDS remain largely unknown.

In the present study, our data revealed that BTZ resistant SKM-1R cells are characterized by upregulation of ERK. These findings indicate that upregulation of ERK activity may be a potential mechanism for BTZ resistance. MEK/ERK pathway is activated in aggressive CKS1B-overexpressing plasma cell myeloma cells [29]. Higher expression levels of p-ERK in human prostate cancer samples were associated with tumor progression [30]. Hyperactivation of the MAPK pathway has also been implicated in the development of neurofibromatosis type 1-associated leukemia [31]. Our findings are consistent with these previous reports. We further showed that resistance to BTZ can be reversed by PD98059 and U0126, suggesting that MEK/ERK pathway may be a potential target for MDS patients who developed resistance to BTZ. Our findings are in line with previous report that ERK inhibitors synergized with BTZ on anticancer effects in medulloblastoma cells [32].

Using the MUTZ-1 cell line, Huang et al found no notable changes in p-ERK1/2 after BTZ treatment [33]. The difference in p-ERK expression in response to BTZ treatment may be explained by the nature of the two different cell lines. MUTZ-1 was isolated from the malignant cells of a 5-year-old girl with MDS [34], whereas SKM-1 was established from leukemic cells of a 76-year-old patient [16]. These two cell lines exhibit different chromosome abnormalities.

Autophagy is a mechanism that degrades dysfunctional cellular components through lysosomes. The activation of autophagy may lead to either cell death or increased survival depending on cell types and conditions. For example, BTZ-induced autophagy led to cell death in MDS/AML cells [21]. Consistent with these previous findings, our present study showed that BTZ induced the conversion of LC3I to LC3II in wild type SKM-1 cells but not in BTZ resistance SKM-1R cells. Our results suggest that autophagic cell death may contribute, at least in part, to BTZ-induced cell cycle arrest and apoptosis.

In some cases, the LC3-I to LC3-II conversion is necessary but not sufficient to trigger cell autophagy. Further studies are needed to ascertain the role of autophagy in BTZ induced cell death by silencing autophagy related proteins such as ATG5, ATG7 and ATG8 in Skm-1 cells.

In conclusion, the present study demonstrates that BTZ induces cell cycle arrest, apoptosis and autophagy in SKM-1 cells. The cytotoxic effects of BTZ appeared to depend, at least in part, on the inhibition of the MEK/ERK pathway. The BTZ resistant SKM-1R cells are characterized by upregulation of ERK. Based on the present observations, a combinational therapy of BTZ and MAPK inhibitors may be an effective complement to current therapeutic approaches for MDS.

## References

[1] San-Miguel JF, Richardson PG, Gunther A, Sezer O, Siegel D, et al (2013) Phase ib study of panobinostat and bortezomib in relapsed or relapsed and refractory multiple myeloma. J Clin Oncol 31: 3696–3703. 24019544 10.1200/JCO.2012.46.7068

[2] Richardson PG, Barlogie B, Berenson J, Singhal S, Jagannath S, et al (2003) A phase 2 study of bortezomib in relapsed, refractory myeloma. N Engl J Med 348: 2609–2617. 12826635 10.1056/NEJMoa030288

[3] Petrucci MT, Giraldo P, Corradini P, Teixeira A, Dimopoulos MA, et al (2013) A prospective, international phase 2 study of bortezomib retreatment in patients with relapsed multiple myeloma. Br J Haematol 160: 649–659. 23293914 10.1111/bjh.12198

[4] Attar EC, Amrein PC, Fraser JW, Fathi AT, McAfee S, et al (2013) Phase I dose escalation study of bortezomib in combination with lenalidomide in patients with myelodysplastic syndromes (MDS) and acute myeloid leukemia (AML). Leuk Res 37: 1016–1020. 23773898 10.1016/j.leukres.2013.05.011 PMC3969839

[5] Howard DS, Liesveld J, Phillips GL 2nd, Hayslip J, Weiss H, et al (2013) A phase I study using bortezomib with weekly idarubicin for treatment of elderly patients with acute myeloid leukemia. Leuk Res. 10.1016/j.leukres.2013.09.003 PMC4025941 24075534

[6] Wang AH, Wei L, Chen L, Zhao SQ, Wu WL, et al (2011) Synergistic effect of bortezomib and valproic acid treatment on the proliferation and apoptosis of acute myeloid leukemia and myelodysplastic syndrome cells. Ann Hematol 90: 917–931. 21340723 10.1007/s00277-011-1175-6

[7] Natarajan-Ame S, Park S, Ades L, Vey N, Guerci-Bresler A, et al (2012) Bortezomib combined with low-dose cytarabine in Intermediate-2 and high risk myelodysplastic syndromes. A phase I/II Study by the GFM. Br J Haematol 158: 232–237. 22571447 10.1111/j.1365-2141.2012.09153.x

[8] Ricciardi MR, McQueen T, Chism D, Milella M, Estey E, et al (2005) Quantitative single cell determination of ERK phosphorylation and regulation in relapsed and refractory primary acute myeloid leukemia. Leukemia 19: 1543–1549. 16001087 10.1038/sj.leu.2403859

[9] Kornblau SM, Womble M, Qiu YH, Jackson CE, Chen W, et al (2006) Simultaneous activation of multiple signal transduction pathways confers poor prognosis in acute myelogenous leukemia. Blood 108: 2358–2365. 16763210 10.1182/blood-2006-02-003475 PMC1895551

[10] Shelton JG, Steelman LS, Abrams SL, Bertrand FE, Franklin RA, et al (2005) The epidermal growth factor receptor gene family as a target for therapeutic intervention in numerous cancers: what’s genetics got to do with it? Expert Opin Ther Targets 9: 1009–1030. 16185155 10.1517/14728222.9.5.1009

[11] Chung E, Hsu CL, Kondo M (2011) Constitutive MAP kinase activation in hematopoietic stem cells induces a myeloproliferative disorder. PLoS One 6: e28350. 22164275 10.1371/journal.pone.0028350 PMC3229546

[12] Naci D, El Azreq MA, Chetoui N, Lauden L, Sigaux F, et al (2012) alpha2beta1 integrin promotes chemoresistance against doxorubicin in cancer cells through extracellular signal-regulated kinase (ERK). J Biol Chem 287: 17065–17076. 22457358 10.1074/jbc.M112.349365 PMC3366820

[13] Fenouille N, Puissant A, Dufies M, Robert G, Jacquel A, et al (2010) Persistent activation of the Fyn/ERK kinase signaling axis mediates imatinib resistance in chronic myelogenous leukemia cells through upregulation of intracellular SPARC. Cancer Res 70: 9659–9670. 21098700 10.1158/0008-5472.CAN-10-2034

[14] Abrams SL, Steelman LS, Shelton JG, Wong EW, Chappell WH, et al (2010) The Raf/MEK/ERK pathway can govern drug resistance, apoptosis and sensitivity to targeted therapy. Cell Cycle 9: 1781–1791. 20436278 10.4161/cc.9.9.11483 PMC3781182

[15] Suzuki M, Abe A, Imagama S, Nomura Y, Tanizaki R, et al (2010) BCR-ABL-independent and RAS/MAPK pathway-dependent form of imatinib resistance in Ph-positive acute lymphoblastic leukemia cell line with activation of EphB4. Eur J Haematol 84: 229–238. 20002159 10.1111/j.1600-0609.2009.01387.x

[16] Nakagawa T, Matozaki S, Murayama T, Nishimura R, Tsutsumi M, et al (1993) Establishment of a leukaemic cell line from a patient with acquisition of chromosomal abnormalities during disease progression in myelodysplastic syndrome. Br J Haematol 85: 469–476. 8136267 10.1111/j.1365-2141.1993.tb03334.x

[17] Lew DJ, Kornbluth S (1996) Regulatory roles of cyclin dependent kinase phosphorylation in cell cycle control. Curr Opin Cell Biol 8: 795–804. 8939679 10.1016/s0955-0674(96)80080-9

[18] Hiraoka Y, Yamada T, Goto M, Das Gupta TK, Chakrabarty AM (2004) Modulation of mammalian cell growth and death by prokaryotic and eukaryotic cytochrome c. Proc Natl Acad Sci U S A 101: 6427–6432. 15082831 10.1073/pnas.0401631101 PMC404061

[19] Manni S, Brancalion A, Mandato E, Tubi LQ, Colpo A, et al (2013) Protein Kinase CK2 Inhibition Down Modulates the NF-kappaB and STAT3 Survival Pathways, Enhances the Cellular Proteotoxic Stress and Synergistically Boosts the Cytotoxic Effect of Bortezomib on Multiple Myeloma and Mantle Cell Lymphoma Cells. PLoS One 8: e75280. 24086494 10.1371/journal.pone.0075280 PMC3785505

[20] Hu J, Van Valckenborgh E, Xu D, Menu E, De Raeve H, et al (2013) Synergistic induction of apoptosis in multiple myeloma cells by bortezomib and hypoxia-activated prodrug TH-302, in vivo and in vitro. Mol Cancer Ther 12: 1763–1773. 23832122 10.1158/1535-7163.MCT-13-0123

[21] Fang J, Rhyasen G, Bolanos L, Rasch C, Varney M, et al (2012) Cytotoxic effects of bortezomib in myelodysplastic syndrome/acute myeloid leukemia depend on autophagy-mediated lysosomal degradation of TRAF6 and repression of PSMA1. Blood 120: 858–867. 22685174 10.1182/blood-2012-02-407999 PMC3412348

[22] Kabeya Y, Mizushima N, Ueno T, Yamamoto A, Kirisako T, et al (2000) LC3, a mammalian homologue of yeast Apg8p, is localized in autophagosome membranes after processing. EMBO J 19: 5720–5728. 11060023 10.1093/emboj/19.21.5720 PMC305793

[23] Oerlemans R, Franke NE, Assaraf YG, Cloos J, van Zantwijk I, et al (2008) Molecular basis of bortezomib resistance: proteasome subunit beta5 (PSMB5) gene mutation and overexpression of PSMB5 protein. Blood 112: 2489–2499. 18565852 10.1182/blood-2007-08-104950

[24] Shuqing L, Jianmin Y, Chongmei H, Hui C, Wang J (2011) Upregulated expression of the PSMB5 gene may contribute to drug resistance in patient with multiple myeloma when treated with bortezomib-based regimen. Exp Hematol 39: 1117–1118. 21920470 10.1016/j.exphem.2011.09.003

[25] Ria R, Catacchio I, Berardi S, De Luisi A, Caivano A, et al (2013) HIF-1alpha of bone marrow endothelial cells implies relapse and drug resistance in patients with multiple myeloma and may act as a therapeutic target. Clin Cancer Res. 10.1158/1078-0432.CCR-13-1950 24297864

[26] Bosman MC, Schuringa JJ, Quax WJ, Vellenga E (2013) Bortezomib sensitivity of acute myeloid leukemia CD34(+) cells can be enhanced by targeting the persisting activity of NF-kappaB and the accumulation of MCL-1. Exp Hematol 41: 530–538 e531. 10.1016/j.exphem.2013.02.002 23416210

[27] Smith AJ, Dai H, Correia C, Takahashi R, Lee SH, et al (2011) Noxa/Bcl-2 protein interactions contribute to bortezomib resistance in human lymphoid cells. J Biol Chem 286: 17682–17692. 21454712 10.1074/jbc.M110.189092 PMC3093844

[28] Shringarpure R, Catley L, Bhole D, Burger R, Podar K, et al (2006) Gene expression analysis of B-lymphoma cells resistant and sensitive to bortezomib. Br J Haematol 134: 145–156. 16846475 10.1111/j.1365-2141.2006.06132.x

[29] Shi L, Wang S, Zangari M, Xu H, Cao TM, et al (2010) Over-expression of CKS1B activates both MEK/ERK and JAK/STAT3 signaling pathways and promotes myeloma cell drug-resistance. Oncotarget 1: 22–33. 20930946 10.18632/oncotarget.105 PMC2949973

[30] Kinkade CW, Castillo-Martin M, Puzio-Kuter A, Yan J, Foster TH, et al (2008) Targeting AKT/mTOR and ERK MAPK signaling inhibits hormone-refractory prostate cancer in a preclinical mouse model. J Clin Invest 118: 3051–3064. 18725989 10.1172/JCI34764 PMC2518074

[31] Staser K, Park SJ, Rhodes SD, Zeng Y, He YZ, et al (2013) Normal hematopoiesis and neurofibromin-deficient myeloproliferative disease require Erk. J Clin Invest 123: 329–334. 23221339 10.1172/JCI66167 PMC3533306

[32] Yang F, Jove V, Chang S, Hedvat M, Liu L, et al (2012) Bortezomib induces apoptosis and growth suppression in human medulloblastoma cells, associated with inhibition of AKT and NF-kB signaling, and synergizes with an ERK inhibitor. Cancer Biol Ther 13: 349–357. 22313636 10.4161/cbt.19239 PMC3341212

[33] Huang J, Ding T, Yang M, Liu H, Sun X, et al (2011) Antitumor activity and drug interactions of proteasome inhibitor Bortezomib in human high-risk myelodysplastic syndrome cells. Int J Hematol 93: 482–493. 21451957 10.1007/s12185-011-0821-z

[34] Steube KG, Gignac SM, Hu ZB, Teepe D, Harms D, et al (1997) In vitro culture studies of childhood myelodysplastic syndrome: establishment of the cell line MUTZ-1. Leuk Lymphoma 25: 345–363. 9168445 10.3109/10428199709114174

